# Supplementary material for: Characterizing cell-type spatial relationships across length scales in spatially resolved omics data
Source: Nat Commun. 2025 Jan 3;16:350. doi: 10.1038/s41467-024-55700-1 (PMC11699133; doi:10.1038/s41467-024-55700-1)
Supplement: Supplementary file 3 — Supplementary Software 1 [file 41467_2024_55700_MOESM3_ESM.zip › CRAWDAD/plots/slideseq/S4_slideseq_purk_ripleys_facet.pdf]

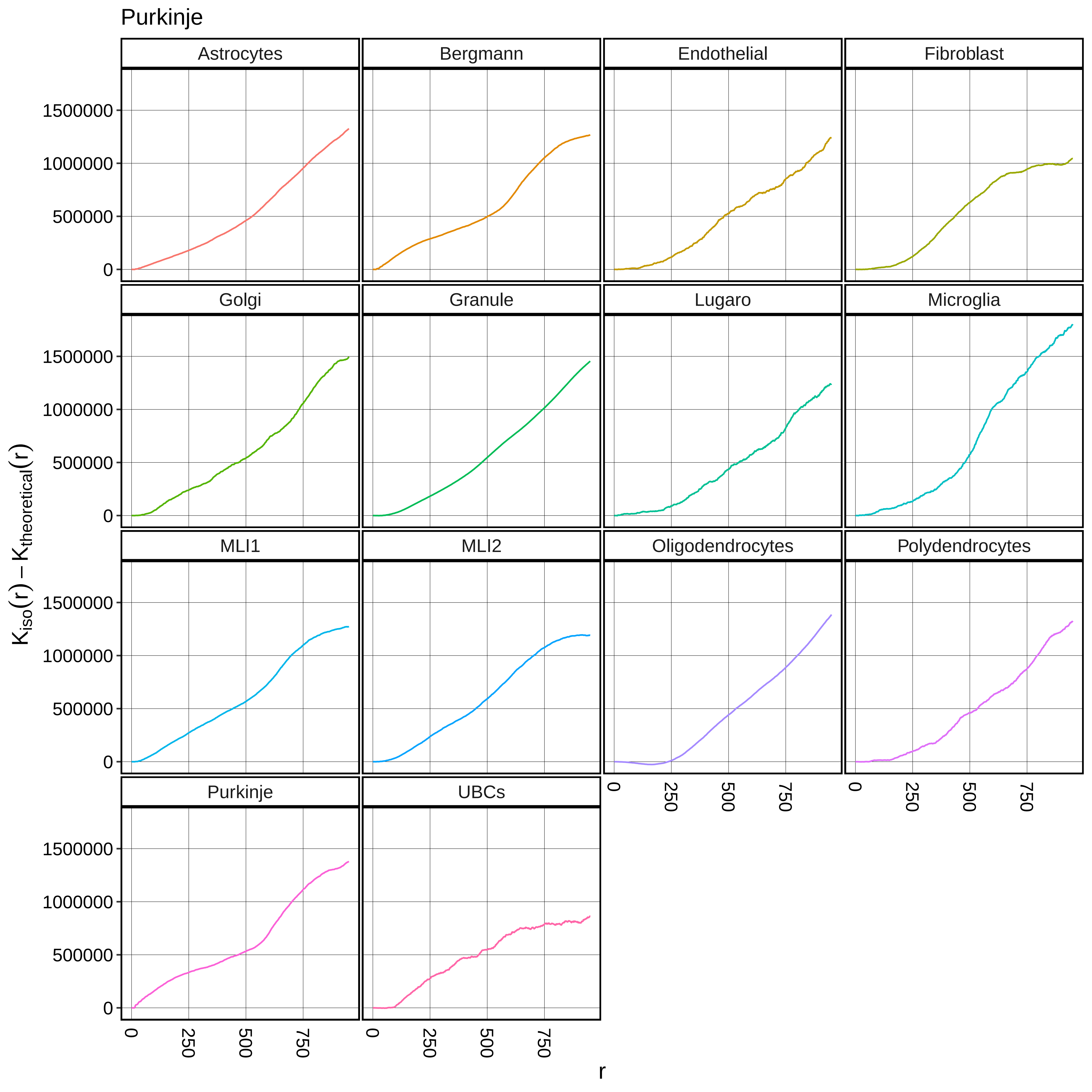

Purkinje
Astrocytes
Bergmann
Endothelial
Fibroblast
Golgi
Granule
Lugaro
Microglia
MLI1
MLI2
Oligodendrocytes
Polydendrocytes
Purkinje
UBCs
0
500000
1000000
1500000
0
250
500
750
$K_{iso}(r) - K_{theoretical}(r)$
r
